# Efficacy of newer versus older antihypertensive drugs in black patients living in sub-Saharan Africa

**DOI:** 10.1038/jhh.2013.56

**Published:** 2013-06-27

**Authors:** J R M'Buyamba-Kabangu, B C Anisiuba, M B Ndiaye, D Lemogoum, L Jacobs, C K Ijoma, L Thijs, H J Boombhi, J Kaptue, P M Kolo, J B Mipinda, C E Osakwe, A Odili, B Ezeala-Adikaibe, S Kingue, B A Omotoso, S A Ba, I I Ulasi, J A Staessen

**Affiliations:** 1 Studies Coordinating Centre, Division of Hypertension and Cardiovascular Rehabilitation, KU Leuven Department of Cardiovascular Sciences, University of Leuven, Leuven, Belgium; 2 Hypertension Unit, Department of Internal Medicine, University of Kinshasa Hospital, Kinshasa, Democratic Republic of Congo; 3 Department of Medicine, College of Medicine, University of Nigeria Teaching Hospital, Enugu, Nigeria; 4 Centre Hospitalier National Aristide Le Dantec, Dakar, Senegal; 5 Douala Cardiovascular Research Institute, Douala School of Medicine, Douala, Cameroon; 6 Yaoundé General Hospital, Yaoundé, Cameroon; 7 Department of Medicine, University of Ilorin Teaching Hospital, Ilorin, Nigeria; 8 Centre Hospitalier de Libreville, Libreville, Gabon; 9 National Biotechnology Development Agency, Medical Biotechnology Department, Abuja, Nigeria; 10 Department of Internal Medicine, College of Health Science, University of Abuja, Abuja, Nigeria; 11 Department of Epidemiology, Maastricht University, Maastricht, The Netherlands

**Keywords:** sub-Saharan Africa, antihypertensive drugs, blacks, randomized clinical trial, special populations

## Abstract

To address the epidemic of hypertension in blacks born and living in sub-Saharan Africa, we compared in a randomised clinical trial (NCT01030458) single-pill combinations of old and new antihypertensive drugs in patients (30–69 years) with uncomplicated hypertension (140–179/90–109 mm Hg). After ⩾4 weeks off treatment, 183 of 294 screened patients were assigned to once daily bisoprolol/hydrochlorothiazide 5/6.25 mg (*n*=89; R) or amlodipine/valsartan 5/160 mg (*n*=94; E) and followed up for 6 months. To control blood pressure (<140/<90 mm Hg), bisoprolol and amlodipine could be doubled (10 mg per day) and α-methyldopa (0.5–2 g per day) added. Sitting blood pressure fell by 19.5/12.0 mm Hg in R patients and by 24.8/13.2 mm Hg in E patients and heart rate decreased by 9.7 beats per minute in R patients with no change in E patients (–0.2 beats per minute). The between-group differences (R minus E) were 5.2 mm Hg (*P*<0.0001) systolic, 1.3 mm Hg (*P*=0.12) diastolic, and 9.6 beats per minute (*P*<0.0001). In 57 R and 67 E patients with data available at all visits, these estimates were 5.5 mm Hg (*P*<0.0001) systolic, 1.8 mm Hg (*P*=0.07) diastolic and 9.8 beats per minute (*P*<0.0001). In R compared with E patients, 45 vs 37% (*P*=0.13) proceeded to the higher dose of randomised treatment and 33 vs 9% (*P*<0.0001) had α-methyldopa added. There were no between-group differences in symptoms except for ankle oedema in E patients (*P*=0.012). In conclusion, new compared with old drugs lowered systolic blood pressure more and therefore controlled hypertension better in native African black patients.

## Introduction

Sub-Saharan Africa is facing an epidemic of cardiovascular disease,^1, 2^ mainly driven by hypertension.^3, 4^ Depending on the age range in published studies,^3^, ^4^, ^5^, ^6^ hypertension currently affects from 30% up to 60% of blacks, born and living in Africa. The 2003 guidelines of the World Health Organization and International Society of Hypertension (WHO/ISH) propose that for the majority of hypertensive patients without a compelling indication for another class of drugs, a low-dose diuretic should be considered as the first choice of therapy on the basis of comparative trial data, availability and cost.^7^ However, recent trials^8, 9^ proved benefit of newer vs older antihypertensive drugs in terms of blood pressure control, reduction of cardiovascular morbidity and mortality, and metabolic side effects. They also indicated that the majority of hypertensive patients require multiple drugs to achieve control.^8, 10^ Modern guidelines for the management of hypertension^11, 12, 13^ endorse the use of single-pill combinations of antihypertensive drugs to initiate treatment. Recommendations for the management of hypertension in native black African patients are to a large extent extrapolations of studies conducted in blacks living in the United States or Europe and do not account for differences in selection in previous generations,^14^ ethnic admixture,^15^ and lifestyle.^16^ We therefore designed the newer versus older antihypertensive agents in African hypertensive patients (NOAAH) to compare in native black African patients a single-pill combination of newer drugs, not involving a diuretic, with a combination of older drugs, including a diuretic.

## Materials and methods

The NOAAH trial was an open, randomised, investigator-led multicentre trial complying with the guidelines for good clinical practice.^17^ The sponsor (Hypertension Unit, University of Kinshasa Hospital, Democratic Republic of Congo) and all participating centres obtained ethical clearance from their local Institutional Review Boards and/or National Regulatory Authorities. Patients provided written or witnessed informed consent at screening.

As outlined in detail in the published protocol,^18^ treatment-naïve or previously treated patients of either sex, aged 30–69 years with uncomplicated grade 1 or grade 2 hypertension and a maximum of two additional risk factors qualified for enrolment. Previously treated patients should not have a compelling indication to continue treatment and should be on a single drug. After a 4-week run-in period off treatment, eligible patients had a sitting blood pressure ranging from 140 to 179 mm Hg systolic or from 90 to 109 mm Hg diastolic, or both. These blood pressure thresholds were averages of three consecutive readings obtained by means of validated^19^ Omron 705IT monitors (Omron Healthcare Co., Ltd., Kyoto, Japan) fitted with a cuff adjusted to arm circumference. To exclude orthostatic hypotension, systolic blood pressure measured immediately after standing up had to be at least 110 mm Hg. In addition to major illness and high cardiovascular risk, the exclusion criteria encompassed atrial fibrillation, electrocardiographic left ventricular hypertrophy with strain pattern, a serum creatinine concentration higher than 1.4 mg dl^−1^ in women or 1.5 mg dl^−1^ in men, overt diabetes mellitus and proteinuria or haematuria on a dipstick test.

The Studies Coordinating Centre (SCC) in Leuven randomised eligible patients, using permuted blocks of four consecutive patients within each centre, to a single-pill combination of 6.25 mg hydrochlorothiazide plus 5 mg bisoprolol (older drugs) or the combination of valsartan 160 mg plus amlodipine 5 mg (newer drugs). To achieve blood pressure control, the study medication could be up titrated to 6.25 mg hydrochlorothiazide plus 10 mg bisoprolol in the reference group and to 160 mg valsartan plus 10 mg amlodipine in the experimental group. If blood pressure remained uncontrolled, doctors could add α-methyldopa up to 2 g per day to the study medication. SCC shipped all medications to the recruiting clinical sites. To assess drug accountability and adherence, each medicine package carried a unique identification number. Patients had to return unused medications at the next visit. Investigators counted the number of unused pills.

Follow-up visits after randomisation took place at 2 weeks and at monthly intervals up to 6 months. The visits at randomisation and 8, 16 and 24 weeks comprised a computerised 12-lead ECG (Cardiax device and software, version 3.50.2, International Medical Equipment Developing Co. Ltd., Budapest, Hungary), an assessment of symptoms and side effects, measurements of haemoglobin, haematocrit, serum sodium, potassium, creatinine and total cholesterol, and blood glucose, and a dipstick test on a fresh urine sample. Patients graded symptoms on a 5-point scale (never, little, moderate, fair and very) by means of a validated questionnaire.^20, 21^ For analysis, the scores of the 34 questions were averaged into an overall score and into organ-specific scores summarising neurosensory, circulatory, gastrointestinal, respiratory and urogenital symptoms (Supplementary Table S1 available in the online data supplement).

The primary outcome was the baseline-adjusted between-group difference in the sitting systolic blood pressure. To demonstrate a 5 mm Hg difference (s.d., 12 mm Hg) with a two-sided *P* of 0.01 and 90% power, 180 randomised patients, 90 per group, were required. Secondary outcomes were time to blood pressure control and incidence of adverse events. Controlled hypertension was a sitting blood pressure below 140 mm Hg systolic and below 90 mm Hg diastolic (average of three consecutive readings).

SAS software (SAS Institute, Cary, NC, USA), version 9.3, was used for database management and statistical analysis. Group statistics include means (s.d.), medians (interquartile range (IQR)) and frequencies (per cent). The main analysis included all randomised patients with at least one follow-up visit according to the intention-to-treat principle. The cohort analysis only included patients who had data at each scheduled visit. Between-group comparisons of means, medians, proportions and Kaplan–Meier survival estimates relied on Student's *t* test, Mann-Whitney's U, the *χ*2 statistic, and the log-rank test, respectively. Treatment effects on continuous variables were analysed using a mixed model with baseline blood pressure and follow-up time as fixed effects and centre as random effect. Cox regression was used to compare time with blood pressure control. Statistical significance was a two-sided *P*-value of 0.05 or less.

## Results

Supplementary Figure S1 and the Supplementary Information show the flow of patients. Among 183 randomised patients, 89 and 94 were allocated to old and new drugs, and 57 and 67 completed the 6-month follow-up. Table 1 shows there were no between-group differences in the baseline characteristics among all analysed patients (*P*⩾0.06) as well as among those in the cohort analysis (*P*⩾0.19), with the exception of body mass index (*P*=0.009) in the cohort analysis. There were no differences among patients included or not included in the cohort analysis (*P*⩾0.17). The study comprised 96 (52.5%) women and 128 (69.9%) treatment-naïve patients. Age (±s.d.) averaged 51.2±9.0 years, ranging from 30.5 to 68.9 years. None of the patients had a fall in systolic blood pressure exceeding 20 mm Hg on standing.

Median follow-up was 24 weeks (IQR, 20–24 weeks) and similar in both groups (*P*=0.24). In patients on old drugs, compared with those assigned new drugs, 45 vs 37% (*P*=0.13) proceeded to the higher dose of randomised treatment and 33 vs 9% (*P*<0.0001) had α-methyldopa added (Supplementary Figure S2). In the cohort analysis, these percentages were 50.9 vs 34.3% (*P*=0.13) and 38.6 vs 7.5% (*P*<0.0001), respectively (Supplementary Figure S3). The median daily dose of α-methyldopa was 0.5 g (IQR, 0.5–1.0 g).

**Figure 1:**
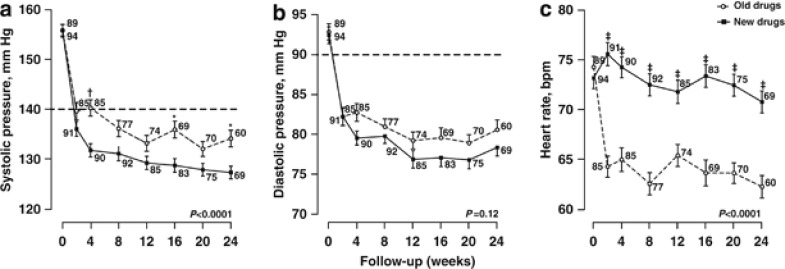
Systolic (**a**) and diastolic (**b**) blood pressures and heart rate (**c**) at randomisation and at various follow-up visits in patients randomised to old drugs (*n*=89) or new drugs (*n*=94). Plotted values are means±s.e. The number of patients contributing to the means is given. *P*-values denote the significance of the between-group differences derived from a mixed model. Significance of the between-group differences at individual visits: *P⩽0.05; †*P*⩽0.01; ‡*P*⩽0.001.

Across all visits, average tablet counts expressed as a percentage of the number to be taken ranged from 86.9 to 94.6% in the old-drug group and from 88.3 to 95.5% in patients allocated to new drugs with no between-group differences (*P*⩾0.07). The overall tablet count averaged (±s.d.) 90±18% (IQR, 90–100). Heart rate decreased (*P*<0.0001) in the patients randomised to old drugs with no changes in the new-drug group (*P*⩾0.12), resulting in a significant (*P*<0.0001) between-group differences at all follow-up visits in all patients (Figure 1) as well as in the cohort (Supplementary Figure S4).

In all participants (Table 2 and Figure 1) and in the cohort (Table 2 and Supplementary Figure S4), systolic and diastolic blood pressures decreased (*P*⩽0.0001) after randomisation, irrespective of whether blood pressure was measured in the sitting or standing position. Sitting systolic blood pressure decreased more in patients randomised to new drugs. In all patients (Table 2), the between-group differences amounted to 5.2 mm Hg (*P*=0.013) at the last visit and to 5.2 mm Hg (*P*<0.0001) for all visits combined. In the cohort analysis, the corresponding differences were 5.4 mm Hg (*P*=0.035) and 5.5 mm Hg (*P*<0.0001), respectively. The Supplementary Material provides the systolic blood pressure differences at successive visits. The baseline-adjusted changes in the sitting diastolic blood pressure (*P*⩾0.32) and in the standing systolic and diastolic blood pressures (*P*⩾0.07) were not different between the study groups (Table 2). The only exception was the standing systolic blood pressure, which was lower on the new than old drugs across all visits in the overall analysis (4.6 mm Hg; CI, 2.1 to 7.1 mm Hg; *P*=0.004; Table 2).

**Figure 2:**
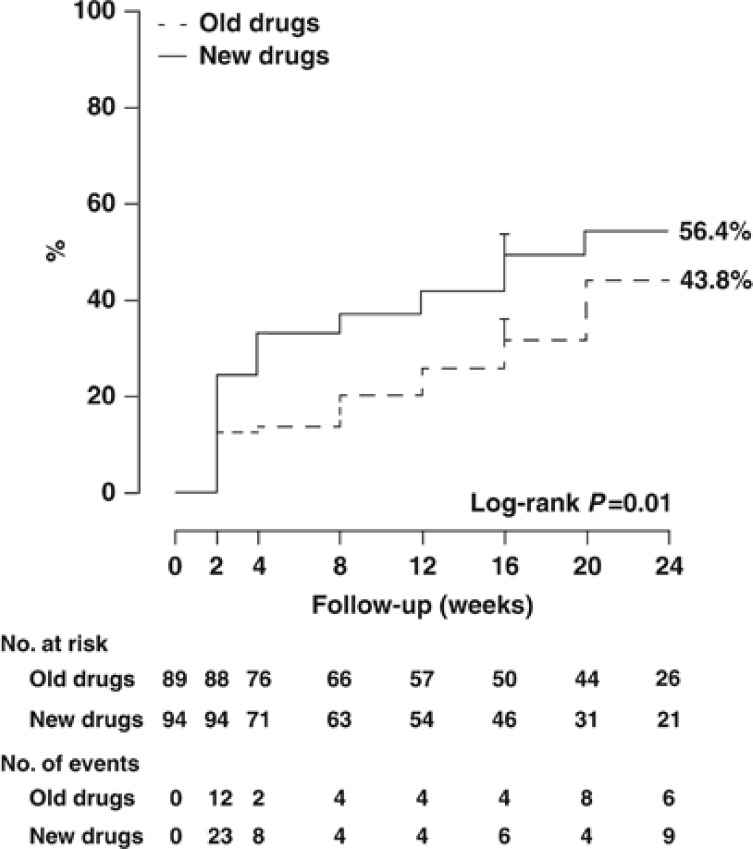
Kaplan–Meier survival function estimates for the probability of reaching blood pressure control in patients randomised to old drugs (*n*=89) or new drugs (*n*=94). Control was a blood pressure lower than 140 mm Hg systolic and lower than 90 mm Hg diastolic. Vertical bars denote the s.e.

At the last available follow-up visit, 58 patients randomised to new drugs (61.7%) and 40 allocated to old drugs (44.9% *P* for between-group difference, 0.023) had reached blood pressure control. Disregarding visits at which patients were taking α-methyldopa, these numbers were 53 (61.6%) and 27 (45.0% *P*=0.047), respectively. The median time interval from randomisation to blood pressure control was 12 weeks (IQR, 4–20) on new drugs and 18 weeks (IQR, 4–24) on old drugs (Figure 2; log-rank *P*=0.011). In Cox regression with adjustments applied for the blood pressure at randomisation, the probability to achieve blood pressure control was 52% greater on new than old drugs (hazard ratio, 1.52; 95% CI, 1.02–2.28; *P*=0.042). Findings in the cohort analysis were confirmatory (Supplementary Figure S5).

During the trial, in all patients and in the cohort, the symptom scores decreased (Supplementary Table S1; *P*⩽0.010) with no between-group difference (*P*⩾0.29). After randomisation, the between-group differences in the organ-specific and individual symptom scores were not significant (*P*⩾0.057), with the exception of ankle oedema that achieved a higher score in patients on new drugs (Supplementary Table S1; *P*=0.012). The within-group changes (*P*⩾0.10) and the between-group differences (*P*⩾0.08) in the haematological and biochemical measurements and in the ECG Cornell index did not reach statistical significance (Supplementary Table S2). Two patients were withdrawn from the trial because of adverse effects (Supplementary Figure S1), one from the old-drug group, because of insomnia and asthenia, and one from the new-drug group, because of bilateral leg oedema. There were no incident cases of diabetes mellitus, gout or hypercholesterolaemia in either treatment group.

## Discussion

The key NOAAH finding was that over 6 months of follow-up the sitting systolic blood pressure decreased by ∼5 mm Hg more on a single-pill combination of new drugs compared with old drugs. Blood pressure control was achieved sooner on new drugs with less need of the addition of α-methyldopa. Over the whole follow-up, the standing systolic blood pressure also decreased by 4.6 mm Hg. In analyses of the blood pressure changes from randomisation to the last follow-up visit, sitting systolic blood pressure was 5.2 mm Hg lower on new drugs, but only 3.9 mm Hg in the standing position (*P*=0.11). Analyses involving blood pressure changes from baseline to the last follow-up visit were confounded by the threefold higher usage of α-methyldopa in the old-drug group. In general, standing blood pressure readings are less standardised than in the sitting position, because of the varying time intervals between the last sitting and the first standing blood pressure measurement. There were no significant between-group differences in the sitting or standing diastolic blood pressures. Adherence was excellent as exemplified by tablet counts and, more objectively, by the lower heart rate on the β-blocker in the old-drug group and the higher score for ankle oedema in patients randomised to amlodipine in the new-drug group. Symptom scores improved during follow-up with no between-group differences, whereas no changes occurred in the Cornell voltage index. Missing data were addressed in two ways: (i) by carrying the last information forward in the intention-to-treat analysis of all randomised particpants and by a cohort analysis, which included only patients with information available at all scheduled visits.

Our current findings are in agreement with a previous trial,^22^ which showed that calcium-channel blockers are the most effective drug class to initiate antihypertensive treatment in South African blacks and that starting with thiazides or converting-enzyme inhibitors more often required combination therapy to control blood pressure. β-blockers without intrinsic sympathomimetic activity, such as bisoprolol, decrease heart rate and cardiac output. The lesser decrease in systolic blood pressure on the old-drug combination is probably the consequence of the β–blocker-induced reduction of heart rate, which is responsible for a later return of the reflected waves in the central arteries during systole and more pronounced systolic augmentation.^23, 24^ Furthermore, under treatment with inhibitors of the renin system, but not under treatment with β-blockers, the structural arteriolar abnormalities associated with hypertension regress. The ensuing reduction of the reflection coefficients likely reduces the amplitude of the backward pressure wave and promotes a decrease of systolic blood pressure and pulse pressure in the brachial artery.^25^ The acute increase in peripheral arterial pressure in response to β–blockade vasoconstriction usually wears off during chronic treatment. This might explain why diastolic blood pressure was similar on old and new drugs.

In several design aspects, the NOAAH trial closely followed current US,^11^ European^12, 13^ and African^26^ guidelines for the management of hypertension. First, for patients with uncomplicated grade 1 or grade 2 hypertension with little added risk, the guidelines propose that lifestyle measures be reinforced for several weeks (grade 2), or even months (grade 1), before antihypertensive drug treatment is initiated. All NOAAH patients received counselling on lifestyle. Second, combination therapy was used to initiate antihypertensive treatment. In single-pill combinations, both components potentiate one another. The advantages of combination therapy^27, 28^ are earlier and tighter blood pressure control than monotherapy or sequential combination therapy; simplification of the therapeutic regimen and therefore better adherence; avoidance of dose-dependent adverse effects experienced with higher doses of single agents; and attenuation of the adverse effects of some agents when used alone. For instance, angiotensin II receptor blockers reduce the prevalence of ankle oedema associated with dihydropyridine calcium-channel blockers.^29^ Third, blacks have a higher sensitivity to salt intake and an impaired ability to excrete ingested salt. This leads to an overall expansion of the intravascular volume. According to the AB/CD algorithm,^12^ both treatment arms of NOAAH included a drug class that addressed the low-renin volume component of hypertension (hydrochlorothiazide and amlodipine) as well as an agent (bisoprolol and valsartan) interfering with the high-renin vasoconstrictor component. Diuretics and calcium-channel blockers potentiate the efficacy of renin system inhibitors in black low-renin patients.^30^

Cost containment is important in the management of common chronic diseases, such as hypertension, especially in resource-poor settings, where out-of-pocket medical expenditure is usual practice. In the countries, in which NOAAH was running, many cheap generics are being sold, however, with minimal quality requirements or even without any regulation via illegal channels. While the older drugs are cheaper, their chronic use contributes to the development of side effects such as the metabolic syndrome, diabetes mellitus, gout and dyslipidaemia. More importantly, treatment with new antihypertensive drugs results in lower morbidity and mortality.^8, 9^ These considerations should be accounted for when current guidelines for the treatment of black hypertensive patients are updated. One limitation of the current study is that it did not include a formal cost-effectiveness analysis.

Over 6 months, the new compared with the old drugs lowered systolic blood pressure ∼5 mm Hg more. If sustained over years, a 5-mm Hg lower systolic blood pressure might be associated with a 20% reduction of cardiovascular mortality and a 30% decrease in major cardiovascular complications.^31^ Furthermore, NOAAH demonstrated that a simple therapeutic regimen consisting of a single-pill combination of either old or new drugs efficiently lowered blood pressure in black patients born and living in sub-Saharan Africa, thereby extending and generalising previous findings in African Americans^32^ and South African blacks.^22^ NOAAH therefore revealed an enormous potential to curb the epidemic of premature cardiovascular mortality and morbidity,^1, 2^ mainly caused by hypertension,^3, 4^ in sub-Saharan Africa. Several African countries, such as Nigeria, implement programmes, in which patients with HIV or tuberculosis are being followed up and treated free of cost. The time might have come to consider similar approaches to screen for hypertension, the leading cause of death in the developing world,^2^ and to provide patients access to antihypertensive treatment at low or no cost.

In addition to its scientific objectives, NOAAH also intended to build capacity among sub-Saharan investigators to conduct randomised clinical trials in the field of cardiovascular medicine. NOAAH showed that conducting such trials in this region of the world met problems related to regulations, logistics, infrastructure and know how. It took 5 years (2008^4^–2013) to proceed from planning to publication. To our knowledge, NOAAH is the first successful randomised clinical trial of antihypertensive treatment in developing countries located in sub-Saharan West Africa. The skills learnt by local investigators should be useful for much more demanding studies in the future. Such trials might have intermediate or hard endpoints rather than change in blood pressure and must address the cost-effectiveness of various pharmacological options to treat the legions of hypertensive patients in sub-Saharan Africa.


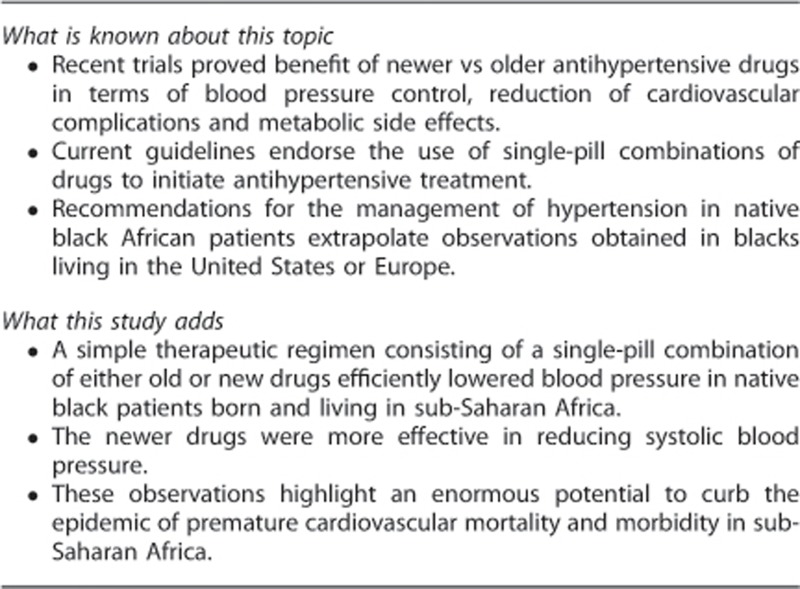


## Figures and Tables

**Table 1:** Baseline characteristics by type of analysis and randomisation group

*Characteristic*	*Type of analysis*			
	*All participants*		*Cohort*	
	*Old*	*New*	*Old*	*New*
*Number (%) with characteristic*				
All patients in category	89	94	57	67
Women	43 (48.3)	53 (56.4)	33 (57.9)	37 (55.2)
Smokers	6 (6.7)	1 (1.1)	1 (1.8)	1 (1.5)
Drinking alcohol	29 (32.6)	27 (28.7)	15 (26.3)	20 (29.9)
Treatment naïve	64 (71.9)	64 (68.1)	39 (68.4)	43 (64.2)
				
*Mean±s.d. of characteristic*				
Age, years	51.2±8.7	51.3±9.3	52.1±7.9	50.9±9.1
Body mass index, kg m^−2^	28.7±4.6	27.6±4.8	29.3±4.5	27.1±4.4*
				
Sitting measurements of				
Systolic pressure, mm Hg	155.9±10.8	155.8±12.7	157.2±11.3	155.0±12.1
Diastolic pressure, mm Hg	92.9±9.7	92.5±10.3	94.0±9.6	92.0±10.3
Heart rate, beats per min	74.3±10.1	73.2±10.4	74.9±10.0	72.6±9.3
				
*Standing measurements of*				
Systolic pressure, mm Hg	156.9±13.8	156.6±14.7	158.2±14.9	155.2±13.2
Diastolic pressure, mm Hg	97.6±10.0	98.8±9.9	98.4±10.2	98.0±10.0
Heart rate, beats per min	81.6±12.2	80.1±12.3	82.2±13.0	80.5±9.5
				
*Measurements on blood*				
Haemoglobin, mg dl^−1^	12.9±1.6	12.8±1.7	12.6±1.7	12.8±1.9
Haematocrit, %	38.9±4.7	39.0±5.5	38.1±4.9	39.0±5.9
Serum creatinine, μmol l^−1^	85.5±24.1	86.9±32.8	85.5±20.7	91.1±36.2
Serum cholesterol, mmol l^−1^	4.9±1.4	4.9±1.1	4.9±1.2	4.8±1.2
Blood glucose, mmol l^−1^	5.1±0.9	5.0±0.7	5.1±0.9	5.0±0.7
ECG Cornell index, mm × msec	1816±833	1817±631	1872±850	1716±604

The analysis of all participants and of the cohort encompasses patients with at least one follow-up visit after randomisation and patients who attended all scheduled visits, respectively. Old and new refer to single-pill combinations of hydrochlorothiazide plus bisoprolol and valsartan plus amlodipine. Measurements of blood pressure are averages of three consecutive readings. In the analysis of all participants, the number of patients with blood samples ranged from 83 to 89 and from 88 to 94 in the old and new drugs groups. In the cohort analysis, these numbers ranged from 52 to 57 and from 61 to 67, respectively. Between-group differences in the baseline characteristics among all patients (*P*⩾0.06) and among those in the cohort analysis (*P*⩾0.19) were not significant with the exception of body mass index in the cohort analysis (*P=0.009). To convert creatinine, cholesterol and glucose from mmol l^−1^ to mg dl^−1^, divide by 88.4, 0.0259 and 0.0555, respectively.

**Table 2:** Changes in blood pressure and heart rate by randomisation group and type of analysis

*Characteristic*	*Type of analysis*							
	*All participants*				*Cohort*			
	*Old*	*New*	Δ *(CI)*	P	*Old*	*New*	Δ *(CI)*	P
Number	89	94			57	67		
*Sitting position*								
*Last visit*								
Systolic pressure, mm Hg	−21.2±1.5*	−26.4±1.5*	−5.2 (−9.3 to−1.1)	0.013	−22.6±1.8*	−28.0±1.7*	−5.4 (−10.3 to−0.4)	0.035
Diastolic pressure, mm Hg	−13.1±1.0*	−13.0±1.2*	−0.1 (−3.0 to 3.1)	0.96	−13.2±1.2*	−14.1±1.3*	−0.8 (−4.5 to 2.8)	0.65
Heart rate, beats per minute	−10.3±1.2*	−1.0±1.1	9.3 (6.0 to 12.6)	<0.0001	−12.5±1.4*	−1.8±1.2	10.7 (7.1 to 14.3)	<0.0001
								
*All visits*								
Systolic pressure, mm Hg	−19.5±1.2*	−24.8±1.2*	−5.2 (−7.7 to−2.9)	<0.0001	−21.6±1.4*	−25.7±1.3*	−5.5 (−8.1 to−2.9)	<0.0001
Diastolic pressure, mm Hg	−12.0±0.8*	−13.2±0.9*	−1.3 (−3.0 to 0.3)	0.12	−12.9±0.9*	−13.9±1.0*	−1.8 (−3.7 to 0.1)	0.073
Heart rate, beats per minute	−9.7±1.0*	0.2±0.9	9.6 (7.7 to 11.5)	<0.0001	−11.3±1.1*	−0.2±1.0	9.8 (7.7 to 11.9)	<0.0001
								
*Standing position*								
*Last visit*								
Systolic pressure, mm Hg	−21.9±1.7*	−25.8±1.7*	−3.9 (−8.6 to 0.8)	0.11	−25.2±2.0*	−26.8±2.0*	−1.6 (−7.3 to 4.1)	0.58
Diastolic pressure, mm Hg	−12.9±1.0*	−14.3±1.1*	−1.5 (−4.4 to 1.4)	0.30	−13.4±1.2*	−14.7± 1.3*	1.3 (−2.2 to 4.8)	0.46
Heart rate, beats per minute	−11.7±1.3*	−0.5±1.2	11.2 (7.6 to 14.8)	<0.0001	−13.5±1.7*	−1.9±1.4	11.6 (7.4 to 15.8)	<0.0001
								
*All visits*								
Systolic pressure, mm Hg	−18.9±1.4*	−23.8±1.3*	−4.6 (−7.1 to−2.1)	0.004	−21.3±1.8*	−24.1±1.5*	−4.6 (−7.2 to−2.0)	0.0008
Diastolic pressure, mm Hg	−12.0±0.8*	−14.1±0.8*	−1.3 (−2.8 to 0.20)	0.092	−12.7±1.0*	−14.4±0.9*	−1.6 (−3.3 to 0.1)	0.072
Heart rate, beats per minute	−11.4±1.2*	0.7±0.9	11.3 (9.2 to 13.3)	<0.0001	−12.9±1.5*	−0.5±0.9	11.3 (9.0 to 13.6)	<0.0001

Abbreviation: CI, confidence interval.

The analysis of all participants and of the cohort encompasses patients with at least one follow-up visit after randomisation and patients who attended all scheduled visits, respectively. Old and new refer to single-pill combinations of hydrochlorothiazide plus bisoprolol and valsartan plus amlodipine. Within-group decreases (follow-up minus baseline) are mean±s.e. Δ (CI) refers to the baseline-adjusted differences (95% confidence interval) of the treatment effects (new minus old). A negative value of Δ (CI) indicates lower values on treatment with new drugs. *P*-values for the between-group differences were computed from *t*-tests for the data at the last visit and from mixed models for all visits combined. An asterisk indicates significance (*P*<0.001) for the within-group change in blood pressure or heart rate.
