# Supplementary material for: Efficacy of newer versus older antihypertensive drugs in black patients living in sub-Saharan Africa
Source: J Hum Hypertens. 2013 Jun 27;27(12):729–35. doi: 10.1038/jhh.2013.56 (PMC3831294; doi:10.1038/jhh.2013.56)
Supplement: Supplementary Information [file jhh201356x1.doc]

**Journal of Human Hypertension**

**Supplementary materials**

Supplementary materials were provided by the authors to give readers additional information about their work.

Supplement to:
M’Buyamba-Kabangu JR, Anisiuba BC, Ndiaye MB, Lemogoum D, Jacob L, Ijoma CK, Thijs L, Boombhi HJ, Kaptue J, Kolo PM, Mipinda JB, Osakwe CE, Odili A, Ezeala-Adikaibe B, Kingue S, Omotoso BA, Ba SA, Ulasi II, and Staessen JA on behalf of the Newer versus Older Antihypertensive Agents in African Hypertensive Patients Trial (NOAAH) Investigators. Efficacy of newer versus older antihypertensive drugs in black patients living in sub-Saharan Africa. *Journal of Human Hypertension* 2013 ...

**Expanded results**

*Flow of patients*— Six centres located in Cameroon (*n*=2), Gabon (*n*=1), Nigeria (*n*=2), and Senegal (*n*=1) enrolled patients. Of 294 screened patients, 271 were enrolled in the run-in period, and 183 were randomized: 89 and 94 to old and new drugs, respectively (Figure S1). The reasons why 88 patients were excluded from randomisation included: blood pressure below (*n*=33) or above (*n*=1) the entry criteria (*n*=34); discontinuation of previous antihypertensive treatment for less than 4 weeks (*n*=7); serum creatinine concentration above 1.4 mg/dl in women or above 1.5 mg/dl in women (*n*=5); overt diabetes mellitus (*n*=4); arrhythmia (*n*=3); informed consent not given (*n*=3); lost to follow-up during the run-in period (*n*=4); or undocumented reasons (*n*=28). In the old-and new-drug groups, 57 and 67 patients completed the 6‑month follow-up; 10 and 9 patients defected from the old- and new-drug groups for undocumented reasons; 22 and 18 left the study, because they had moved (*n*=9 and 3), changed working hours (*n*=4 and 3), or withdrew consent (*n*=3 and 2). Doctors withdrew 6 and 10 patients from the old- and new-drug groups, because of logistical reasons (*n*=5 and 9) or adverse effects (*n*=1 and 1). Logistical reasons included delayed replenishment of the local supply of study medications and internet or computer failures at the local centres.

*Blood pressure effects at successive visits*—Figure 1 shows that in all patients the baseline-adjusted between-group differences in systolic blood pressure were significant at 1 month (8.7 mm Hg; 95% confidence interval [CI], 13.4–4.3 mm Hg; P=0.0001), 4 months (5.7 mm Hg; CI, 10.6 to 0.8 mm Hg; P=0.021), and at 6 months (4.8 mm Hg; 9.6 to 0.1 mm Hg; P=0.043). In the cohort analysis (Figure S4), at the same time points, these estimates were 5.2 mm Hg (CI, 10.1 to 0.3 mm Hg; P=0.038), 6.8 mm Hg (CI, 12.3 to 1.3 mm Hg; P=0.015) and 5.4 mm Hg (CI, 10.3 to 0.4 mm Hg; P=0.035).

*Cholesterol at baseline and last follow-up*—Patients were subdivided into those with normal, mildly elevated and high serum cholesterol concentration at baseline: (i) >200 mg/dl (<5.18 mmol/l); (ii) 200-239 mg/dl (5.18-6.19 mmol/l); and (iii) ≥240 mg/dl (≥6.20 mmol/l). The number (%) of patients with normal, mildly elevated or high cholesterol in the old-drug group was 60 (68.2%), 18 (20.4%), and 10 (11.4%); in the new‑drug group, these proportions were 55 (61.5%), 26 (28.6%), and 9 (9.9%). The difference at baseline between the 2 groups was not significant (*P*=0.89). At the end of follow-up in 57 patients randomised to old drugs, these numbers were: 36 (63.2%), 14 (24.5% and 7 (12.3% and in 67 patients allocated new drugs 47 (70.2%), 14 (20.9%, and 8.9%, respectively. The within-group changes in the cholesterol classes were not statistically significant (*P*≥0.69). The number of patients progressing to a higher cholesterol class was 12 (21.4%) of 56 at risk in the old‑drug group and 12 (18.5%) of 65 at risk in the new‑drug group (P for between-group difference, 0.82).

**Table S1.**  Changes in symptom scores by type of analysis and randomisation group

| *Characteristic* |  | *Type of analysis* | | | | | | | | |
| --- | --- | --- | --- | --- | --- | --- | --- | --- | --- | --- |
|  |  | *All participants* | | | |  | *Cohort* | | | |
|  |  | Old | New | Δ (CI) | *P* |  | Old | New | Δ (CI) | *P* |
| Nº of patients |  | 77 | 92 |  |  |  | 52 | 56 |  |  |
| All symptoms |  | -0.10±0.02‡ | -0.08±0.02† | -0.015 (-0.050 to 0.020) | 0.40 |  | -0.14±0.03‡ | -0.10±0.03* | -0.021 (-0.059 to 0.017) | 0.29 |
| Neurosensory |  | -0.19±0.04‡ | -0.17±0.04‡ | -0.010 (-0.065 to 0.044) | 0.71 |  | -0.22±0.04‡ | -0.20±0.06† | -0.020 (-0.040 to 0.080) | 0.52 |
| Circulatory |  | -0.14±0.03‡ | -0.10±0.03* | 0.047 (-0.001 to 0.095) | 0.057 |  | -0.18±0.04‡ | -0.12±0.05* | -0.057 ( -0.110 to -0.003) | 0.042 |
| Ankle oedema |  | -0.04±0.03 | 0.11±0.05 | 0.120 (0.027 to 0.210) | 0.012 |  | -0.06±0.05 | 0.10±0.04* | 0.053 (-0.029 to 0.130) | 0.21 |
| Gastrointestinal |  | -0.06±0.02* | -0.05±0.02* | -0.015 (-0.038 to 0.017) | 0.46 |  | -0.09±0.03† | -0.03±0.02 | -0.025 (-0.003 to 0.054) | 0.084 |
| Respiratory |  | -0.10±0.05 | -0.06±0.03 | 0.031 (-0.027 to 0.089) | 0.30 |  | -0.14±0.06 | -0.08±0.04 | -0.024 (-0.099 to 0.051) | 0.53 |
| Urogenital |  | 0.04±0.02 | 0.06±0.03 | 0.021 (-0.040 to 0.081) | 0.50 |  | 0.01±0.03 | 0.00±0.02 | -0.012 (-0.049 to 0.074) | 0.69 |

The analysis of all participants and of the cohort encompasses patients with at least one follow-up visit after randomisation and patients who attended all scheduled visits, respectively. Within-group decreases (follow-up minus baseline) are mean ± SE. Δ (CI) refers to the baseline-adjusted differences (95% confidence interval) of the treatment effects (new minus old). A negative value of Δ (CI) indicates lower values on treatment with new drugs. *P* values were computed using all available data from mixed models. In the analysis of all participants, the number of patients with a questionnaire available ranged from 60 to 77 and from 67 to 92 in the old and new drugs groups, respectively. * *P*≤0·05; † *P*≤0·01; ‡ *P*≤0·001 for the within-group changes in symptom scores.

**Neurosensory symptoms:** feeling tense, exhausted, shivery, depressed, being active, concentration disturbances, disturbed vision, spots before eyes, headache, sleeplessness, and vivid dreams; **circulatory symptoms:** dizziness, unsteady upon standing, palpitations, hot flushes, skin rashes, and ankle oedema; **urogenital symptoms:** nightly visits to the toilet, difficulty in passing urine, pain or burning when passing urine, and decreased interest in sex; **gastrointestinal symptoms:** dry mouth, diarrhoea, constipation, nausea, stomach ache, and vomiting; **respiratory symptoms:** breathlessness, blocked or running nose, cough, and expectoration.

**Table S2.**  Changes in symptom scores by type of analysis and randomisation group

| *Characteristic* |  | *Type of analysis* | | | | | | | | |
| --- | --- | --- | --- | --- | --- | --- | --- | --- | --- | --- |
|  |  | *Overall* | | | |  | *Cohort* | | | |
|  |  | Old | New | Δ (CI) | *P* |  | Old | New | Δ (CI) | *P* |
| Nº of patients |  | 77 | 92 |  |  |  | 52 | 56 |  |  |
| Haemoglobin, mg/dl |  | -0.15±0.14 | -0.31±0.14 | 0.22 (-0.08 to 0.52) | 0.15 |  | -0.18±0.17 | -0.28±0.16 | -0.07 (-0.42 to 0.27) | 0.68 |
| Haematocrit, % |  | -0.07±0.39 | -0.98±0.46 | 0.85 (-0.10 to 1.81) | 0.08 |  | -0.25±0.45 | -0.96±0.53 | -0.46 (-1.48 to 0.57) | 0.38 |
| Serum creatinine, μmol/l |  | -3.21±2.82 | -4.41±3.38 | -1.15 (-6.96 to 4.66) | 0.70 |  | 0.87±2.70 | -6.20±4.30 | -3.90 (-9.75 to 2.97) | 0.30 |
| Serum cholesterol, mmol/l |  | -0.20±0.16 | -0.25±0.10 | 0.17 (-0.10 to 0.43) | 0.23 |  | -0.20±0.14 | -0.30±0.13 | -0.16 (-0.45 to 0.13) | 0.28 |
| Blood glucose, mmol/l |  | -0.04±0.09 | 0.20±0.12 | 0.04 (-0.16 to 0.24) | 0.68 |  | 0.04±0.10 | 0.02±0.09 | -0.05 (-0.24 to 0.13) | 0.57 |
| ECG Cornell index, mm × sec |  | 105±70 | -37±77 | -158 (-334 to 19) | 0.08 |  | 65±83 | -50±88 | -94 (-115 to 303) | 0.38 |

The analysis of all participants and of the cohort encompasses patients with at least one follow-up visit after randomisation and patients who attended all scheduled visits, respectively. Within-group decreases (baseline minus follow-up) are mean ± SE. Δ (CI) refers to the baseline-adjusted differences (95% confidence interval) of the treatment effects (new minus old). A negative value of Δ (CI) indicates lower values on treatment with new drugs. In the analysis of all patients, the number of patients with blood samples available ranged from 83 to 89 and from 88 to 94 in the old and new drugs groups, respectively. In the cohort analysis, these numbers ranged from 52 to 57 and from 61 to 67, respectively. *P* values were computed using all available data from mixed models. There were no significant within-group changes in the measurements (*P*≥0.10 for all participants and *P*≥0.22 for the cohort).

**
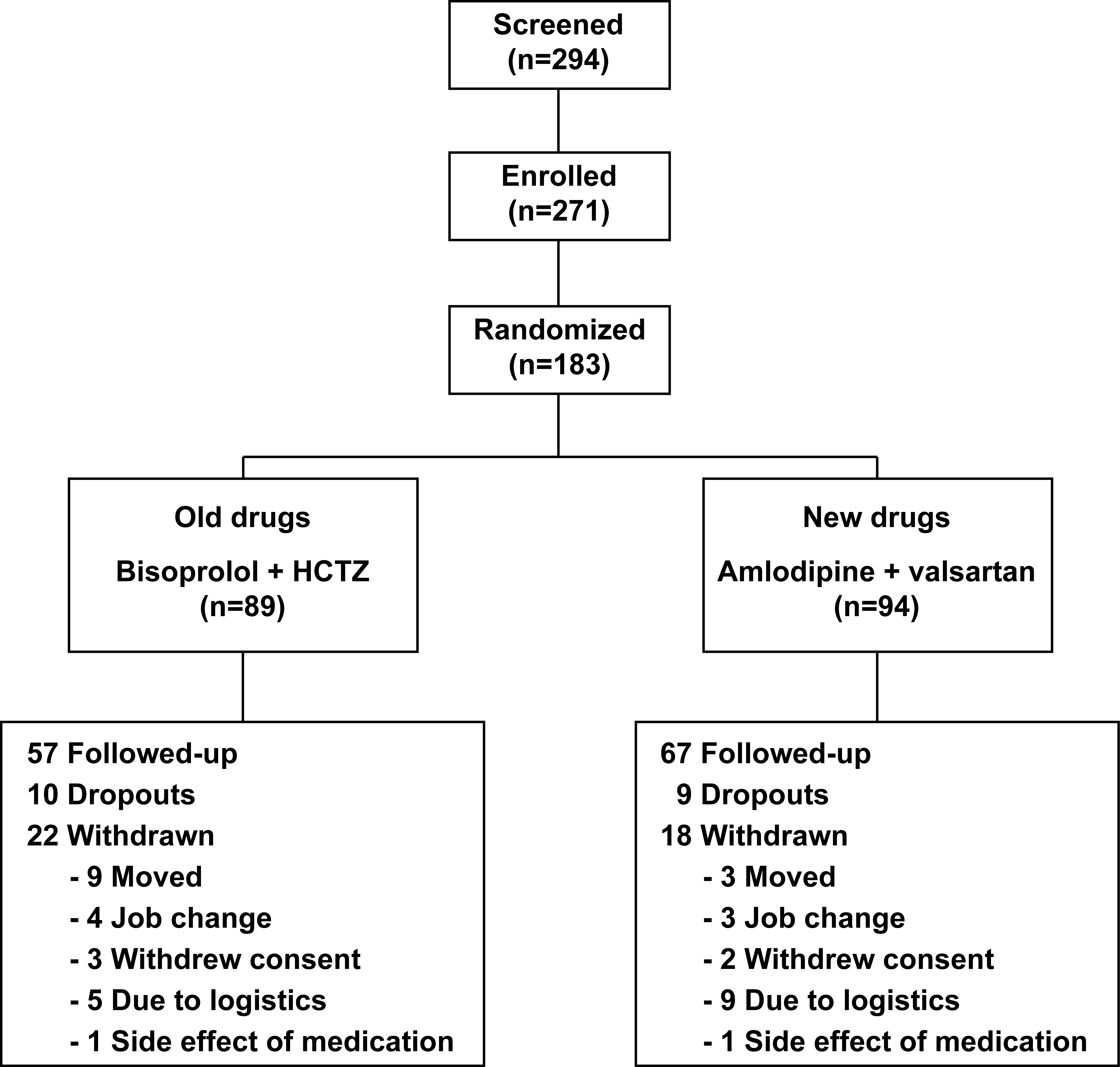
**

**Figure S1.** Flow diagram of patients. Logistical reasons included delayed replenishment of the local supply of study medications and internet or computer failures at local centres. HCTZ indicates hydrochlorothiazide.


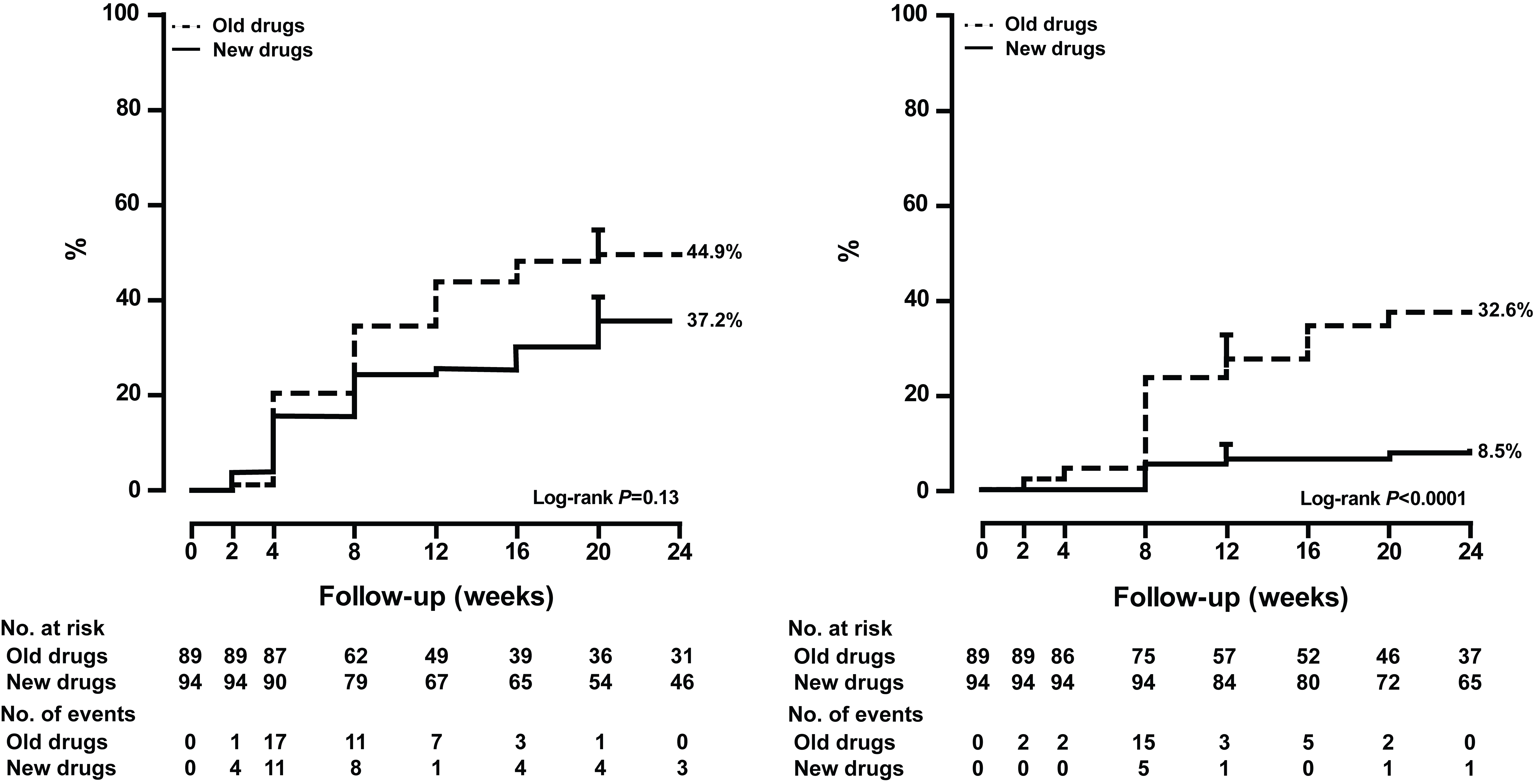


**Figure S2**. Kaplan-Myer survival function estimates for the probability of proceeding to the higher dose study medication (A) or having α‑methyldopa added (B) in patients randomised to old drugs (*n*=89) or new drugs (*n*=94). Vertical bars denote the SE.


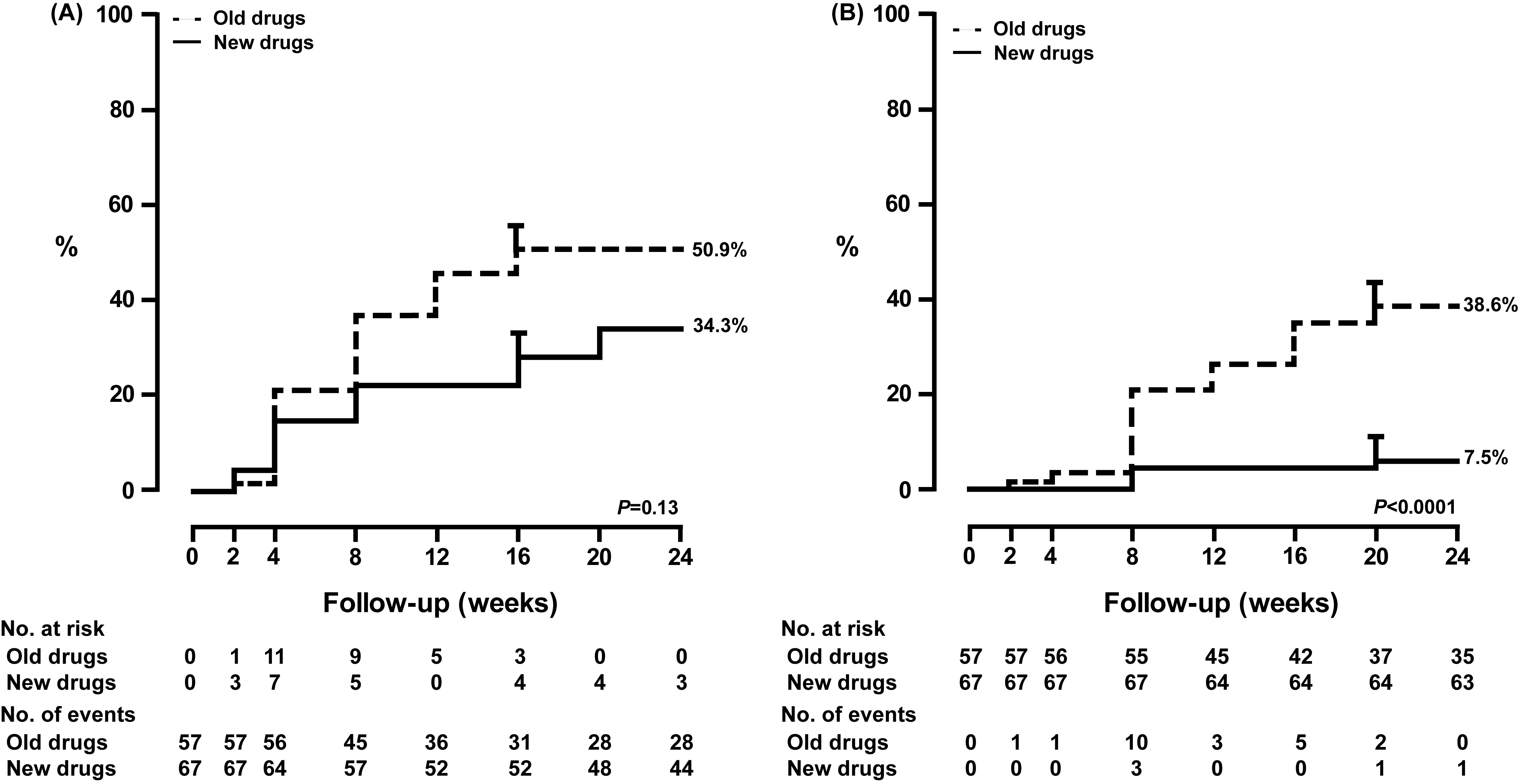


**Figure S3.** Kaplan-Meier survival function estimates for the probability of proceeding to the higher dose study medication (A) or having α‑methyldopa added (B). This cohort analysis included 57 and 67 patients randomised to old or new drugs with data available at each follow-up visit. Vertical bars denote the SE. *P* values are for the significance of the log-rank test.


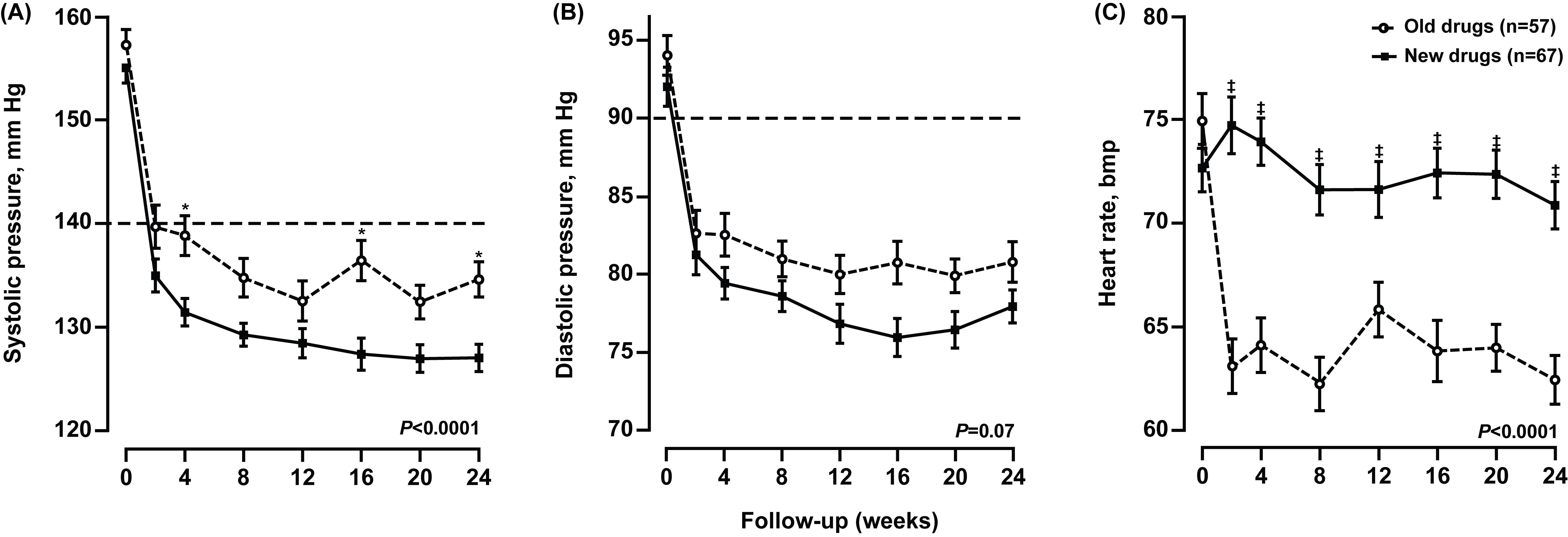


**Figure S4.** Systolic (A) and diastolic (B) blood pressure and heart rate (C) at randomisation and at various follow-up visits. This cohort analysis included 57 and 67 patients randomised to old or new drugs with data available at each follow-up visit. Plotted values are means ± SE. *P* values denote the overall significance of the between-group difference derived from a mixed model. Significance of the between-group differences at individual visits: * *P*≤0.05; ‡ *P*≤0.001.


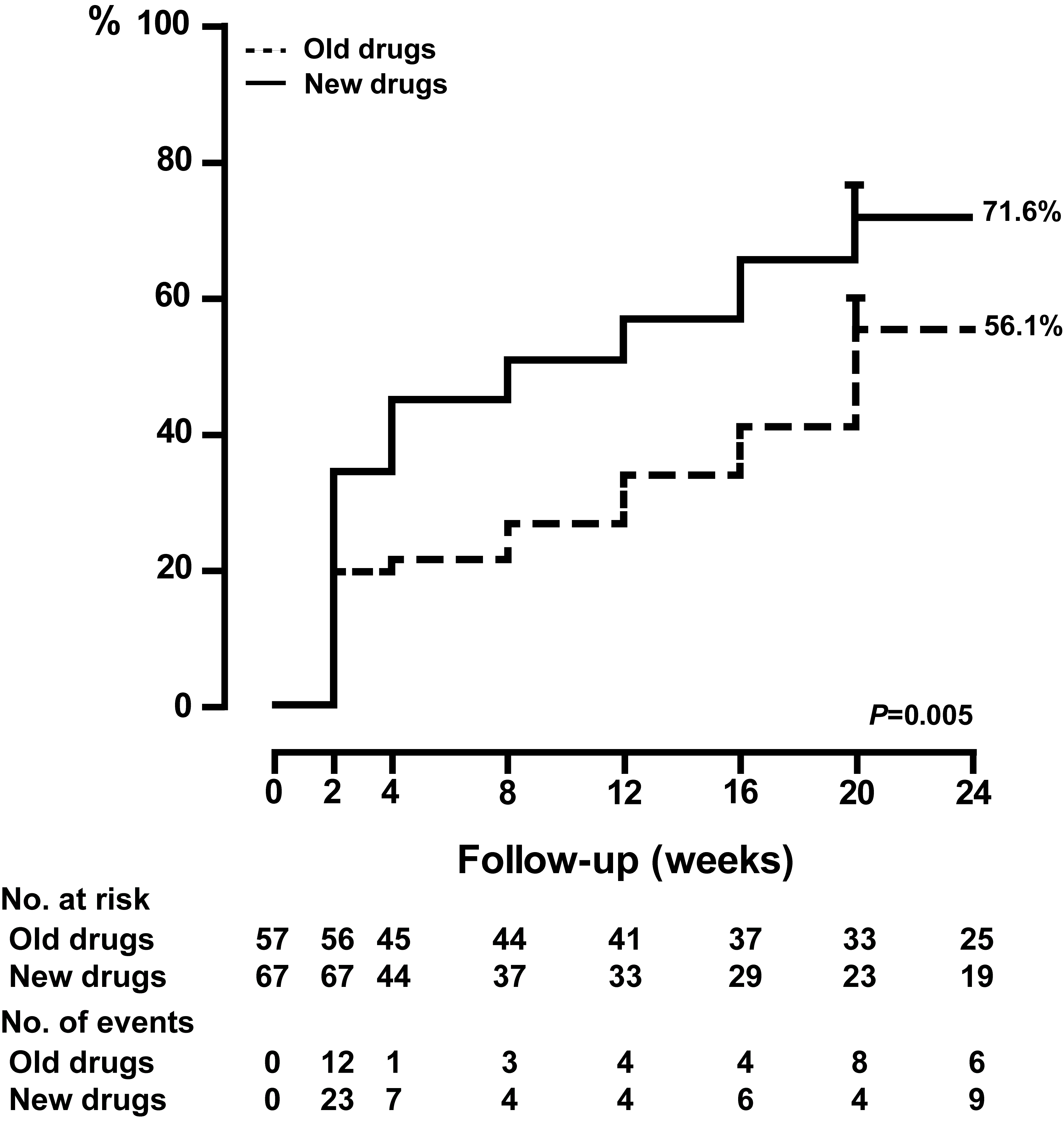


**Figure S5.** Kaplan-Meier survival function estimates for the probability of reaching blood pressure control. This cohort analysis included 57 and 67 patients randomized to old or new drugs with data available at each follow-up visit. Control was a blood pressure lower than 140 mm Hg systolic and lower than 90 mm Hg diastolic. Vertical bars denote the SE. *P* value is for the significance of the log-rank test.
